# The nutrition-based comprehensive intervention study on childhood obesity in China (NISCOC): a randomised cluster controlled trial

**DOI:** 10.1186/1471-2458-10-229

**Published:** 2010-05-02

**Authors:** Yanping Li, Xiaoqi Hu, Qian Zhang, Ailing Liu, Hongyun Fang, Linan Hao, Yifan Duan, Haiquan Xu, Xianwen Shang, Jun Ma, Guifa Xu, Lin Du, Ying Li, Hongwei Guo, Tingyu Li, Guansheng Ma

**Affiliations:** 1 National Institute For Nutrition And Food Safety, Chinese Center For Disease Control And Prevention, Beijing, China; 2 Institute Of Children And Adolescent Health, Peking University Health Science Center, Beijing, China; 3 Department Of Public Health, Shandong University, Shandong Province, China; 4 Guangzhou Center For Disease Control And Prevention, Guangdong Province, China; 5 Harbin Medical University, Heilongjiang Province, China; 6 School Of Public Health, Fudan University, Shanghai, China; 7 Chongqing Children's Hospital, Chongqing, China

## Abstract

**Background:**

Childhood obesity and its related metabolic and psychological abnormalities are becoming serious health problems in China. Effective, feasible and practical interventions should be developed in order to prevent the childhood obesity and its related early onset of clinical cardiovascular diseases. The objective of this paper is to describe the design of a multi-centred random controlled school-based clinical intervention for childhood obesity in China. The secondary objective is to compare the cost-effectiveness of the comprehensive intervention strategy with two other interventions, one only focuses on nutrition education, the other only focuses on physical activity.

**Methods/Design:**

The study is designed as a multi-centred randomised controlled trial, which included 6 centres located in Beijing, Shanghai, Chongqing, Shandong province, Heilongjiang province and Guangdong province. Both nutrition education (special developed carton style nutrition education handbook) and physical activity intervention (Happy 10 program) will be applied in all intervention schools of 5 cities except Beijing. In Beijing, nutrition education intervention will be applied in 3 schools and physical activity intervention among another 3 schools. A total of 9750 primary students (grade 1 to grade 5, aged 7-13 years) will participate in baseline and intervention measurements, including weight, height, waist circumference, body composition (bioelectrical impendence device), physical fitness, 3 days dietary record, physical activity questionnaire, blood pressure, plasma glucose and plasma lipid profiles. Data concerning investments will be collected in our study, including costs in staff training, intervention materials, teachers and school input and supervising related expenditure.

**Discussion:**

Present study is the first and biggest multi-center comprehensive childhood obesity intervention study in China. Should the study produce comprehensive results, the intervention strategies would justify a national school-based program to prevent childhood obesity in China.

**Trial Registration:**

Chinese clinical trial registry (Primary registry in the WHO registry network) Identifier: ChiCTR-TRC-00000402

## Background

China is facing with the serious obesity epidemic, both in adults and children [1-4], and it even starts to shift to the poor [5], where the public health still focused on under-nutrition. Applying the WHO growth reference (2007) [6], the overweight/obesity and stunting prevalence of Chinese children and adolescents aged 5-19 years was 6.2% and 13.8% [7], respectively. Nearly 30% (China National reference: BMI ≥ 24 kg/m^2^) [1,8] or 25% (International standard: BMI ≥ 25 kg/m^2^) [3,9] adults were overweight or obese in China. Furthermore, risk factors for cardiovascular disease tend to cluster in childhood and are strongly associated with obesity [10-12]. We need to find efficient intervention and prevention strategies to encourage people to adopt a healthy lifestyle, or we may face a higher rate of death, disease, and disability and the related costs [13,14]. The indirect effects of obesity and obesity-related dietary and physical activity patterns reached 3.4% of gross national product (GNP) in 2000 and was projected to reach 8.73 percent in 2025 [15]. The need for effective prevention of overweight and obesity is generally considered to be urgent.

Prevention and treatment of obesity and overweight may be somewhat easier in children than in adults because children are still growing in height [16] and their behaviours and lifestyle are still under developing. Related to the increased energy needs during growth, a child can achieve reductions in adiposity without reducing energy intake. However, when develop the intervention, caution should be taken that to ensure the normal growth and development of children [17,18]. Nutrition education and physical activity intervention have been considerable potential as a means of prevention of weight gain in general population. School provides an ideal opportunity in term of both physical and social environment for preventing and treating obesity. Therefore, school-based obesity interventions have been applied worldwide as well as in China. Previous pilot study and effectiveness evaluation study of school-based nutrition and/or physical activity intervention programs in China [19] indicated that childhood obesity interventions were feasible, effective and sustainable in Chinese students. However, whether it would be successful when expand in large scale (from more regions to national-wide) remains unclear. Furthermore, no information is available about the cost-effectiveness of different interventions in China, which crucial for the policy makers. Therefore, we are conducting a school-based intervention study in 6 provinces in China from the beginning of 2009 to the end of 2010.

The objective of this paper is to describe the design of a multi-centred random controlled school-based clinical intervention for childhood obesity in China. The main purpose of the intervention study is to evaluate the feasibility and effectiveness of the comprehensive intervention program for childhood obesity which combined nutrition education and physical activity interventions. The second purpose of the intervention study is to compare the cost-effectiveness of the comprehensive intervention strategy with two other interventions, one only focuses on nutrition education, the other only focuses on physical activity. The final aim of the study is to raise the awareness of the national childhood obesity prevention and control policy recommendations.

## Methods/Design

### Study design

The study is designed as a multi-centred randomiised controlled trial, which included 6 centres located in Beijing, Shanghai, Chongqing, Shandong province, Heilongjiang province and Guangdong province. Both nutrition education and physical activity intervention will be applied in all intervention schools of 5 cities, while in Beijing, nutrition education intervention will be applied in 3 schools and physical activity intervention among the other 3 schools. Nothing will be done in control schools.

The Ethical Review Committee of National institute for nutrition and food safety, Chinese center for disease control and prevention approved the study design, protocols, procedures and informed consent. Participation is voluntary. All participant students and their parents signed informed consent.

### Participants

**Figure 1:**
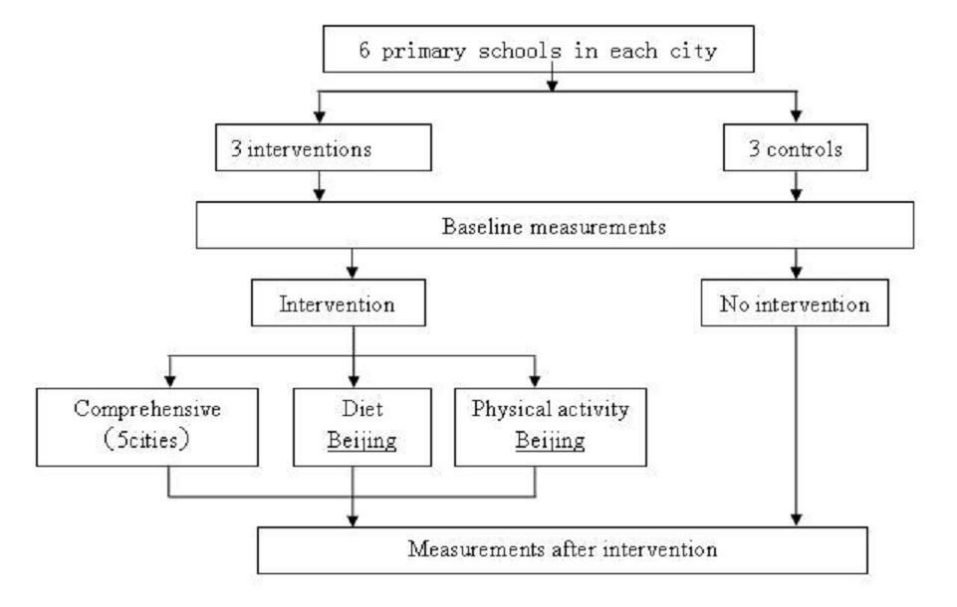
**The study design in each center**.

A total of 9750 primary students (grade 1 to grade 5, aged 7-13 years) will participate in baseline and intervention researches of questionnaire, physical examination, and blood biochemistry and so on. The study design in each center is presented in Figure 1. The method of two-step cluster sampling will be adopted. The first step is that randomly selected 6 schools from each centre assigned to either intervention (3 schools) or control condition (3 schools). The second step is that randomly choose 2 classes from each grade, totally, around 250 subjects will be randomly selected from each school.

### Sample size calculations

The variable used for the calculation of sample size is the BMI changes. To detect a difference of 0.7 kg/m^2 ^of BMI changes between the intervention and control groups, the minimum number required would be 3 schools in each center with 250 students in each school. The intraclass correlation is assumed to be 0.05. The sample size of 7500 students from 30 schools located in 5 centers has 90% power to detect a mean between-group difference in BMI of 0.7 units with an effect size of 0.64. Statistical significance is set at 5% (two-sided). Another 2250 students from 9 schools are randomly chosen only for cost-effect analysis, 3 schools for nutrition intervention, 3 schools for physical activity intervention and another 3 schools are treated as control schools.

### Intervention

Three interventions are included in present study: nutrition education, physical activity intervention and comprehensive intervention, the detail strategies are shown in Table 1.

**Table 1:** Contents of interventions

Group	Interventions
control group	No intervention
Physical activity intervention group	1. "Happy 10" campaign(2 times/day 10 minutes/time or 1 time/day 20 minutes/time) 2. Parents, health workers/health education teacher: "happy 10" training.
Nutrition intervention group	1. Students take part in "Nutrition and Health" classes 2. Parents participate. in "nutrition and health" classes. Give parents corresponding publicity materials. 3. Teacher, health/health education teacher participate in "nutrition and health" classes. Give them corresponding publicity materials. 4. School canteen managers, operators, competent leadership and staff room participate in "nutrition and health" classes. 5. Give them corresponding publicity materials.
Comprehensive (Nutrition & Physical activity) intervention group	Happy 10+nutrition education 1. Students take part in "Nutrition and Health" classes 2. "Happy 10" campaign(2 times/day 10 minutes/time or 1 time/day 20 minutes/time 3. Parents participate. in "nutrition and health" classes. Give parents corresponding publicity materials. 4. Teacher, health/health education teacher participate in "nutrition and health" classes. Give them corresponding publicity materials. And give them a "happy 10" training. 5. School canteen managers, operators, competent leadership and staff room participate in "nutrition and health" classes.

#### Nutrition education

The main nutrition education handbook [20] is developed in a style of carton, developed by department of student nutrition, National institute of nutrition and food safety, China CDC. Carton pamphlet will be distributed to all students in intervention schools. Special lectures will be given for 6 times for students and two times for parent. The menu for students of school lunch cafeteria will be evaluated, after that, specific nutrition improvement will be suggested accordingly. Meanwhile, the nutrition education will be provided to students, parents, health workers and teachers.

#### Physical activity

The Happy 10 program [21] is developed as a classroom-based intervention to promote physical activity among primary school students. The program provided a variety of safe, moderate, age- and space-appropriate exercises. Teaching materials included activity cards, video demonstrations, tracking posters, and stickers. Each activity card introduces one exercise and explains how to perform it. The videos show students from the pilot study performing the activities. Teachers could either demonstrate the activity or show it on a video. The tracking poster and stickers are used to illustrate the progress of each class. Meanwhile, the education about physical activity will be provided to students, parents, health workers and teachers.

#### Comprehensive intervention

The comprehensive intervention is a combination of nutrition and physical activity interventions.

### Staff Training

Staffs from National Institute for Nutrition and Food Safety, Chinese CDC had trained the study group members from the cooperation center for five days. Teachers, usually classroom tutors and/or health educators had attended a two-days training session conducted by the staff of their center with the training slides and videos provided by Chinese CDC. They had learned how to integrate the program into the school curriculum, and how to perform the activities. Slides and videos about nutrition, childhood obesity, risk factors, health consequences, and prevention were prepared by Chinese CDC and provided to school teachers. Teachers modelled the lessons to ensure that they understood the recommended techniques and strategies for implementation.

### Outcome measures

Measurements had been collected in the summers of 2009 (baseline) and will be collected again in summer 2010 (intervention). Children fasted the night before and were measured the next morning by trained research staff. Consistent assessment methods were used throughout the study. The research staffs who conducted the measurement were blinded to the intervention assignment. A brief description of all outcome measurements was shown in Table 2.

**Table 2:** Description of outcomes

Name of Outcome	Category of Outcome	Description
Physical examination	Primary outcome	Height, weight, waist circumference, blood pressure
Body Composition	Primary outcome	Bioelectrical impedance, single-standard Water Law
Blood biochemical indices	Primary outcome	Blood glucose, blood cholesterol, blood triglycerides, blood high-density lipoprotein, low density lipoprotein in blood, blood insulin
Dietary intake situations	Primary outcome	24-hour record of 3 days dietary questionnaire
Physical activities	Primary outcome	Seven days of physical activity recall questionnaire, wearing the energy monitoring device, a 24-hour physical activity record sheet
Physical measurements	Primary outcome	Standing long jump, 50 m, 50 m × 8 from the run
School basic conditions	Secondary outcome	Number of students, the class distribution of the floor, the school's facilities and equipment, sports arrangements, nutrition-related staffing, students in the school dining situation
Obesity-related knowledge, attitudes and practices	Primary outcome	Primary school students questionnaire, parents questionnaire, teacher questionnaire, food service personnel questionnaire
Cost	Primary outcome	Cost related to intervention material, training, supervisor, teachers time input, transportation,

#### Weight, height and BMI

Height was measured to an accuracy of 1 mm with a free-standing stadiometer mounted on a rigid tripod. Fasting body weight was measured to the nearest 0.1 kg on a digital scale. BMI was calculated as weight in kilograms divided by height in meters squared (kg/m^2^). The BMI z score, based on age and gender, was calculated for each student using WHO growth references http://www.who.int/growthref/who2007_bmi_for_age/en/index.html. Each participant was classified into 1 of 4 weight categories: underweight (BMI z score < -2 SD), normal weight (BMI z score between -2 SD and 1 SD), overweight (BMI z score between 1 SD and 2 SD), and obese (BMI z score > 2 SD).

#### Waist circumference

Waist circumference was measured mid-way between the lower rib margin and the iliac crest with flexible anthropometric tape. The waist circumference was measured twice to the nearest 0.5 cm. If the variation between these two measurements was greater than 2 cm, a third measurement was taken and the mean was calculated by using the two closest measurements.

#### Body composition

Body composition was measured using a single frequency (50 Hz) hand to foot bioelectrical impendence device (ImpDF50; Impedimed Pty Ltd., Qld, Australia). The bioimpedance measurement required careful placement of four electrodes on the hand and foot. Resistance (R) and reactance (Xc) were determined. Body impedance was calculated as the square root of (R^2^+Xc^2^). Fat free mass (FFM), fat mass, and percent body fat were calculated using the prediction equations suggested by Deurenberg et al [22].

#### Blood pressure

Blood pressure was measured in the supine position using a mercury sphygmomanometer by trained nurses with at least a 10-min rest period before the measurement. Three measurements were taken from all the subjects at 2-min intervals, and the average of the last two measurements was used. These were recorded to the nearest 2 mmHg.

#### Glucose and lipid profiles

Blood samples were taken in the morning after an overnight fast. Plasma glucose was determined in duplicate by a glucose-oxidase method (Daiichi Pharmaceutical Co., Ltd, Tokyo, Japan) within 4 h after a fasting blood sample was obtained. Total cholesterol (TC), triglycerides (TG), LDL cholesterol (LDL) and HDL cholesterol (HDL) were determined by enzymatic methods using commercial kits (Daiichi Pharmaceutical Co., Ltd, Tokyo, Japan). Serum insulin concentrations were determined by radioimmunoassay (RIA).

#### Physical activity

Information on physical activity patterns was collected using a validated 7-day physical activity questionnaire which was interview-administrated for students of grade 2, and self-administrated for students of grade 3-5. The average energy expenditure and duration of total physical activity per day were calculated from the questionnaire [23].

#### Diet

Information on food intake was collected using the 24-hour dietary recall method for three consecutive days (two weekdays and one weekend day) by trained interviewers.

#### Physical fitness

Test results of standing-board jump, 50 meters speed run and 50 meters × 8 shuttle run were used to reflect the physical fitness of the subjects. The distance of standing-board jump which is an index of strength of muscle was measured to the nearest 0.1 cm using the unified and standard band tapes. The time of 50 meters speed run (an index of speed of muscle) and 50 meters × 8 shuttle run (an index of endurance of muscle) was measured to the nearest 0.1 s using the unified and standard (CASIO) HS-70W stopwatch. All the measures were taken by trained physical education teachers.

#### Cost of intervention

Data concerning costs will be collected in our study, including costs in staff training, intervention materials, teachers and school input and supervising fee at baseline and intervention from the summer of 2009 (baseline) to the summer of 2010 (intervention) regarding implement and management of this study. The cost-effectiveness and cost-benefit will be economically analyzed to compare the three strategies, nutrition education intervention, physical activity intervention and the comprehensive intervention.

### Analysis strategy

Initially, the descriptive statistics will be calculated for all variables considered. Anthropometric measurements at baseline will be compared between intervention and control groups using multivariate regression analysis with age and sex in the model. The effects of intervention will be analyzed using mixed procedure, with BMI changes from baseline as the primary outcome variable. The fixed effects include baseline BMI, age, sex, and the intervention group. The schools within center will be treated as a random effect variable. Similar mixed models will be constructed for other outcome variables, including weight, height, BMI z score, fat free mass, fat mass, percent body fat, physical fitness, as well as the chronic disease factors including glucose, insulin, TG, TC, HDL, LDL and blood pressure.

The intervention effect on body composition will be compared according to the real diet and physical activity changes while the intervention effect on chronic disease factors will also be compared between students whose BMI z score increased or not.

Both cost-effectiveness and cost-benefit will be economically analyzed to compare the three strategies, including nutrition education intervention, physical activity intervention and the comprehensive intervention.

## Discussion and Conclusions

Childhood obesity and its related metabolic and psychological abnormalities are becoming serious health problems in China [1-5]. Effective interventions are needed to prevent the childhood obesity and its related early onset of clinical cardiovascular diseases [24], which would be the earlier the better. Evidence-based intervention strategy with good feasibility and effectiveness and best cost-effective were the necessary conditions for a good national application [25].

The biggest challenge for the large scale population study is no one know what will happened during the long period that may be out of control, to weak the intervention effect. For example, H1N1 spread in the second half year of 2009, just at the beginning of the intervention study. Many schools refused the staff members from our study group to enter the schools for supervising the intervention implementation, which at least in part, weak the intervention effect. Another fact that we have to consider is that two times Happy 10 activity per school day do not absolutely add to the normal physical activity of the students but somehow replace some kinds of activities, which also weak our intervention effect from the theoretical level. So we insist that the Happy 10 program should be conducted in the otherwise sedentary activity period. Our main target population is primary students who are not responsible for food purchase and food preparation, which in turn may limit their food-choice behavior. That means even they learn the relative nutrition knowledge from our nutrition education intervention, they may not change what they eat. So we try our best to involve their parents into our intervention study, including but not limited to send them nutrition education bulletin.

Present NISCOC study has both strengths and weaknesses. The NISCOC study develops the first and biggest multi-center comprehensive childhood obesity intervention study. The large sample size provided sufficient power to detect a relatively small effect. The high participant rate of Chinese students reduces some potential bias. Our outcome measures include both changes in body composition and chronic diseases risk factors. We collected the baseline information of body composition and blood pressure of 9867 students (a little bit more than the sample size because more students in some classes and all want to participant in physical measurements). Among them, more than 8500 students gave blood samples, which provide sufficient power to study the intervention benefit for chronic diseases. The limitations of present study include the accuracy of diet and physical activity information because there is no very accurate diet and physical activity measurements that are feasible for large field survey in the young children. Another limitation is the compliance of the students and teachers. We try to improve the supervising strategies to confirm the interventions will be fully implemented by frequent visiting without notice to intervention school. We will also start some competing activities and some seminars for experience changes between centers and schools.

In summary, the program is easy to implement and well-accepted by the schools, teachers, and students as shown by the pilot study [21]. Should the study produce comprehensive results, the comprehensive intervention strategies would justify a national school-based program to prevent childhood obesity in China.

## Competing interests

The authors declare that they have no competing interests.

## Authors' contributions

GM is the principle investigator and XH is the main coordinator of the project. GM and YL were responsible for the proposal design and funding application. GM, YL, XH, QZ, AL, HF, LH, YD, HX, XS participated in the development of the protocol and were all involved in the monitoring and supervising of the intervention study. YL, HF, LH, HX and XS draft the manuscript of present paper. JM, GX, LD, YL, HG and TL were principle investigator of their center and responsible for the implication of the study. All authors were involved in the manuscript revision and have approved this final version.

## Pre-publication history

The pre-publication history for this paper can be accessed here:

http://www.biomedcentral.com/1471-2458/10/229/prepub
